# The attenuated Pseudorabies virus vaccine Bartha K61 induces a weak cellular immunity: implications for the development of PRV-vectored vaccines

**DOI:** 10.3389/fimmu.2024.1489268

**Published:** 2024-12-23

**Authors:** Gang Xing, Hui Li, Chenhe Lu, Haimin Li, Yulan Jin, Yan Yan, Shaobin Shang, Jiyong Zhou

**Affiliations:** ^1^ MOA Key Laboratory of Animal Virology, Zhejiang University Center for Veterinary Sciences, Hangzhou, China; ^2^ Institute of Comparative Medicine, College of Veterinary Medicine, Yangzhou University, Yangzhou, China; ^3^ Jiangsu Co-Innovation Center for Prevention and Control of Important Animal Infectious Diseases and Zoonosis, Yangzhou University, Yangzhou, China

**Keywords:** Pseudorabies virus, live attenuated vaccines, Bartha K61, cellular immunity, T cells

## Abstract

Pseudorabies virus (PRV), causing Aujeszky’s disease in swine, has important economic impact on the pig industry in China and even poses a threat to public health. Although this disease has been controlled by vaccination with PRV live attenuated vaccines (LAVs), the potency of PRV LAVs in inducing cellular immunity has not been well characterized. In this study, using PRV Bartha K61 strain (BK61), the most-used PRV LAVs, as a model, we re-examined the cellular immune response elicited by the BK61 in mice and pigs by multicolor flow cytometry. We found that phenotypic activation of T cells, NK cells and B cells was hardly detected after vaccination. However, antigen-specific IFN-γ-producing CD4 T cells rather than CD8 T cells were dominantly detected but at low frequency upon restimulation with live BK61 virus. These BK61-specific CD4 T cells are also able to simultaneously produce TNF-α and IL-2, showing characteristics of multifunctional T cells. However, BK61-specific CD4 T cells showed weak secondary response upon challenge with PRV DX strain. Further vaccination with PRV-infected dendritic cells (DCs) transiently increased the percentage of IFN-γ-positive CD4 and CD8 T cells but eventually restored to low frequency and did not improve the protective efficacy of BK61 against challenge, suggesting that PRV BK61 induced a relatively weak cellular immunity that could not be overcome by the DC vaccination. Similar immune responses were also observed following vaccination with another PRV LAV HD/c in mice and pigs, suggesting that this may be an intrinsic drawback of PRV LAVs in inducing cellular immunity. Our results demonstrated that PRV LAVs elicited a CD4 Th1-biased weak cellular immunity which is implicative for the development of PRV-vectored vaccine.

## Introduction

Pseudorabies virus (PRV) is the causative agent of Aujeszky’s disease in swine, causing respiratory disease, abortion, and neurological disorders, and has substantial economic impact on the pig industry, especially in China (1) and even poses a threat to public health due to its transmission to human (2, 3). Since 2011, highly virulent PRV variants have emerged in Bartha K61-vaccinated swine farms in many regions of China (4, 5). The virus belongs to members of the genus Varicellovirus in alphaherpesvirus subfamily, along with several human pathogens such as herpes simplex virus 1 (HSV-1), HSV-2, and varicella-zoster virus (VZV) (6). In addition to the unique natural host of pigs, PRV are able to infect most of mammals, including rodents, dogs, cattle, and horses (7), exhibiting a marked neurotropism by invading the peripheral nervous system (PNS) and occasionally the central nervous system (CNS) of their host species, leading to a lethal and systemic inflammatory response in mice (8).

Aujeszky’s disease in pigs has been well controlled by the use of vaccination with inactivated and live attenuated vaccines (LAVs), especially, by those genetically engineered LAVs lacking virulence-determining gene, for instance, the Bartha K61 strain (7). As virus-vectored vaccines are generally believed to be superior to inactivated vaccines in eliciting both antibody responses and cellular immune responses (9). PRV has been developed into a useful vaccine vector for integrating and expressing foreign proteins for vaccine development (7, 10). By deleting virulence factors, for instance, glycoprotein E (gE), glycoprotein I (gI) and Thymidine kinase (TK), that are non-essential for viral replication and inserting foreign genes from different swine pathogens, recombinant PRV-vectored vaccines have been developed against Porcine circovirus type 2 (11–13), Classical swine fever virus (14), Porcine respiratory and reproductive virus (10), Swine influenza virus and African swine fever virus (15, 16). However, the immune properties of PRV as a viral vector have not been fully addressed, which may restrain its applications.

Both humoral and cellular immune responses have been implicated after PRV infection and/or vaccination (17–20). In detail, antibodies-mediated neutralization, antibody-dependent cell-mediated cytotoxicity, and complement-mediated lysis of PRV-infected target cells have been detected in PRV-infected or vaccinated pigs (18, 19). While neutralizing antibody-mediated protection against PRV was well recognized (7, 21), the cellular immune responses to PRV after infection or vaccination were not fully understood. Early studies showed that PRV-immune swine lymphocytes proliferate and secrete lymphokines upon *in vitro* restimulation with live or inactivated PRV (20, 22) and non-major histocompatibility complex (MHC)-restricted cytotoxicity against PRV-infected and uninfected cells has also been detected in pigs after infection or vaccination (19). PRV-specific lymphocytes or cytotoxic lymphocytes can be detected in the blood and draining lymph node after immunization (23, 24), which included IFN-γ-producing CD4^+^/CD6^+^/CD8^+^ memory T helper cells and CD4^−^/CD6^+^/CD8^+bright^ MHC-restricted cytotoxic lymphocytes (22, 25). Further studies showed that gene-depleted PRV mutants were able to induce glycoprotein C-specific cytotoxic T lymphocytes (CTL) in DBA/2 mice and pigs (25, 26) and MHC II-restricted CD4^+^CD8^+^ memory T helper cells in miniature pigs that were able to help the secretion of PRV-specific immunoglobulins by PRV-primed B cells (27). However, vaccine-induced T cell-mediated immunity seemed to just play a role in early protection against PRV in pigs (28) and was unable to provide long-term protection by immunization once (29). By *in vivo* depleting CD4 and CD8 T cells as well as IFN-γ in mice, Bianchi et al. demonstrated that both IFN-γ and CD4 T cells play an important role in conferring protection against lethal PRV infection (30, 31), whereas CD8 T cells response was barely detected and appeared to be non-essential for immune protection (30). Although these studies demonstrated that cellular immunity was induced by different PRV LAVs, the phenotype, functional changes and the magnitude of distinct T cell subsets have not been revealed following PRV vaccination.

In this study, using PRV LAV Bartha-K61 and HD/c as a model, we further characterized the cellular immune response in mice and pigs after immunization. We found that phenotypic activation of T cells, NK cells and B cells was hardly detected after vaccination, but antigen-specific cytokine-producing CD4 T cells were dominantly detected at low frequency. However, PRV-specific CD4 T cells have weak memory response upon challenge and the weak cellular immunity elicited by BK61 could not be enhanced by dendritic cell vaccination, suggesting that there are intrinsic drawbacks and common characteristics for PRV LAVs in inducing Th1-biased immune response.

## Materials and methods

### Ethics statement

This study was carried out in accordance with the recommendations in the Guide for the Care and Use of Laboratory Animals of Zhejiang University. All animal experiments were approved by the Animal Welfare and Ethics Committee at the College of animal science of Zhejiang University with reference number ZJU20230315.

### Animal, cell lines and virus strain

Six to eight-week-old wild-type C57BL/6 mice were purchased from the Center of Comparative Medicine at Yangzhou University. Three to four-week-old healthy piglets were purchased from Kairong biotechnology company (Suzhou, China). All pigs were detected negative for antibodies against PRV-gB and gE antigen, PRRSV, ASFV and CSFV and nucleic acid of PRV, PRRSV, PCV2, CSFV and ASFV. Human embryonic kidney cells (HEK293T) and PCV-free PK-15 cells (ATCC-CCL-33) were cultured in Dulbecco’s modified Eagle’s medium (DMEM; Gibco, America) supplemented with 10% heat-inactivated fetal bovine serum (FBS; Gibco, America). Classical PRV vaccine strain Bartha-K61 (GenBank accession no. JF797217.1) was gifted by Prof. Song Gao at Yangzhou University. Virulent PRV type II strain DX and an attenuated PRV vaccine strain HD/c (gE/TK deletion) was kept in our laboratory (21). All the viruses were propagated in PK-15 cells, titrated, aliquoted in stocks and stored in -80°C in our lab.

### Animal experiments

A total of ninety female C57BL/6 mice were divided into two groups, with 45 mice in each group. One group was intramuscularly and subcutaneously immunized with 1×10^5^ TCID^50^ PRV Bartha K61 in 100μl DMEM (BK61 imm). This dose of Bartha K61 was relatively unlethal to C57BL/6 mice. Another group was mock immunized with 100μl phosphate-buffered saline (PBS). At indicated time-points after immunization, five mice from each group (n=25) were sacrificed, spleens and lymph nodes were harvested for single cell isolation and quantification of viral loads, respectively. Serum samples were collected by tail vein at days 7, 14, and 28 post immunization to determine the antibody titers. At day 30 after immunization, all the remaining mice from both groups (n=20 per group) were challenged with 5×10^3^ TCID^50^ virulent PRV DX strain. At indicated time-points after challenge, mice from each group (n=4-5) were sacrificed, spleens were harvested for single cell preparation and viral load quantification again. Five mice from each group were spared for recording the survival rate after challenge.

For pig study, ten piglets were divided into two groups. One group (n=5) was immunized intramuscularly at the neck with 2 ml of 10^7.5^ TCID_50_/ml PRV HD/c. The other group was immunized with equal volume of DMEM medium. Periphery blood from each pig was collected at 7, 14 and 21 days after immunization. The isolation of PBMCs and intracellular straining of cytokines in T cells were performed and analyzed by flow cytometry as our previous studies (32, 33).

### Primary cell preparation

Single-cell suspensions from spleen and lymph nodes were prepared as previously described (33, 34). Briefly, tissues were mechanically disrupted and pushed through a 70 μm nylon cell strainer and the cells were resuspended in 10 ml PBS containing 2% fetal bovine serum (FBS). Red blood cells were removed from single cell suspensions by hypotonic lysis with Gey’s solution. Cells were counted using a hemocytometer and the final cell concentration was adjusted to 2 × 10^7^ cells/mL before cell surface staining.

### Dendritic cell differentiation and immunization

Bone marrow-derived dendritic cells (BMDCs) were derived from mouse bone marrow progenitors using GM-CSF and IL-4 (PeproTech, Rocky Hill, NJ) at a final concentration of 10 ng/mL and 2 ng/mL, respectively, as previously described (35). At day 6 of culture, BMDCs were harvested and pulsed with inactivated PRV Bartha K61 (iBK61) or infected with live Bartha K61 (BK61) at a multiplicity of infection (MOI) of 3 for 18 h or indicated length of time. iBK61- or BK61-pulsed BMDCs were washed twice and used as stimulators to activate T cells isolated from PRV-immunized and unimmunized mice.

For DC immunization, BMDCs harvested at day 6 of culture were plated in six-well plate with 6.1×10^6^ cells per well, live PRV Bartha K61 was added up to 1×10^6^ TCID_50_ per mL (MOI=0.5) and infected for overnight, then BK61-infected BMDCs were harvested and washed twice, and a number of 2.17×10^6^ cells was sampled and lysed to confirm the infection rate by IFA on PK-15 cells. 5×10^5^ BK61-infected BMDCs (around 1×10^4.6^ TCID_50_ PRV) were used to immunize mice. A total of eighty female C57BL/6 mice were divided into four groups (DMEM, BK61, DC alone and BK61-DCs, respectively), with 20 mice in each group. Group 1 and 2 were intramuscularly immunized with DMEM and 1×10^5^ TCID^50^ PRV Bartha K61 each mouse (BK61), respectively. Group 3 was intraperitoneally immunized with 5×10^5^ uninfected BMDCs each mouse (DC alone). Group 4 was intraperitoneally immunized with 5×10^5^ BK61-infected BMDCs each mouse containing around 1×10^4.6^ TCID_50_ PRV Bartha K61 (BK61-DCs). At indicated time-points after immunization, mice were sacrificed, spleens were harvested for single cell isolation. At day 30 after immunization, five mice from each group were challenged and the survival rate of each group was recorded.

### Immunophenotypic analysis by flow cytometry

Anti-mouse monoclonal antibodies (mAb) against CD8α (53–6.7), CD44 (1M7), CD62L (MEL14), CD127 (A7R34), KLRG1 (2F1/KLRG1), B220 (RA36B2), CD4 (GK 1.5), TCRβ (H57-597), NK1.1 (PK136), TCRγδ (GL-3) and CD69 (H1.2F3) with different fluorochrome conjugate, were purchased either from BioLegend or BD Bioscience (San Diego, CA). Surface marker staining of cells was carried out as our previous reports (36). For detecting the early activation of distinct immune cells after immunization, 2×10^6^ cells were plated into 96-well V-bottom plates and incubated with 2.4G2 Fcγ RII/RIII blocking mAb for 15 min, then stained for 30 min on ice with 50 μL antibody cocktail containing fluorochrome-conjugated mAbs against CD69, CD8α B220, CD4, TCRβ, NK1.1 and TCRγδ. Afterward, cells were washed and resuspended in 200 μL PBS for flow cytometric analysis.

For defining memory T cells, 2×10^6^ splenocytes were plated and stained for 30 min on ice with 50 μL antibody cocktail containing different fluorochrome-labeled mAbs against markers TCRβ, CD8α, CD44, CD62L, CD127, KLRG1 and CD4. Thereafter, cells were washed and resuspended in 200 μL PBS for flow cytometric analysis. Flow cytometry was performed with a FACS CantoII (BD Biosciences, San Jose, CA) and analyzed using FlowJo software (Tree Star, Ashland, OR).

### Intracellular cytokine staining

2×10^6^ cells were plated into 96 round-bottom-well plates and stimulated with or without live BK61 at MOI of 1 or inactivated BK61-pulsed DCs for 18h. Brefeldin A (Sigma, St. Louis, MO) was added for the last 6 h of co-culture at a final concentration of 10 μg/ml. After incubation, cells were harvested and stained for cell surface makers TCRβ, CD8α, and CD4 as above-mentioned protocol, and then fixed with 4% paraformaldehyde for 10 min and permeabilized twice by Fixation/Permeabilization solution (BD Biosciences) following the manufacturer’s protocol. Subsequently, cells were stained with APC-conjugated anti-IFN-γ (XMG1.2, Biolegend), FITC-conjugated anti-TNF-α (MP6-XT22, Biolegend) and PE-conjugated anti-IL-2 mAbs (JES6-5H4, Biolegend). Flow cytometry was performed as described above.

### Statistical analysis

Statistical analyses were performed using Prism software 5.0 (GraphPad, La Jolla, CA). When comparing experimental values from two groups of mice, two-tailed student’s t-tests were used. When comparing experimental values from multiple groups, one-way ANOVA Bonferroni post-tests were used. Statistically significant differences are noted (****P < 0.0001; ***P < 0.001; **P < 0.01; *P < 0.05).

## Results

### Vaccination with PRV Bartha K61 induced no detectable phenotypic activation of NK cells, B cells and T cells

**Figure 1:**
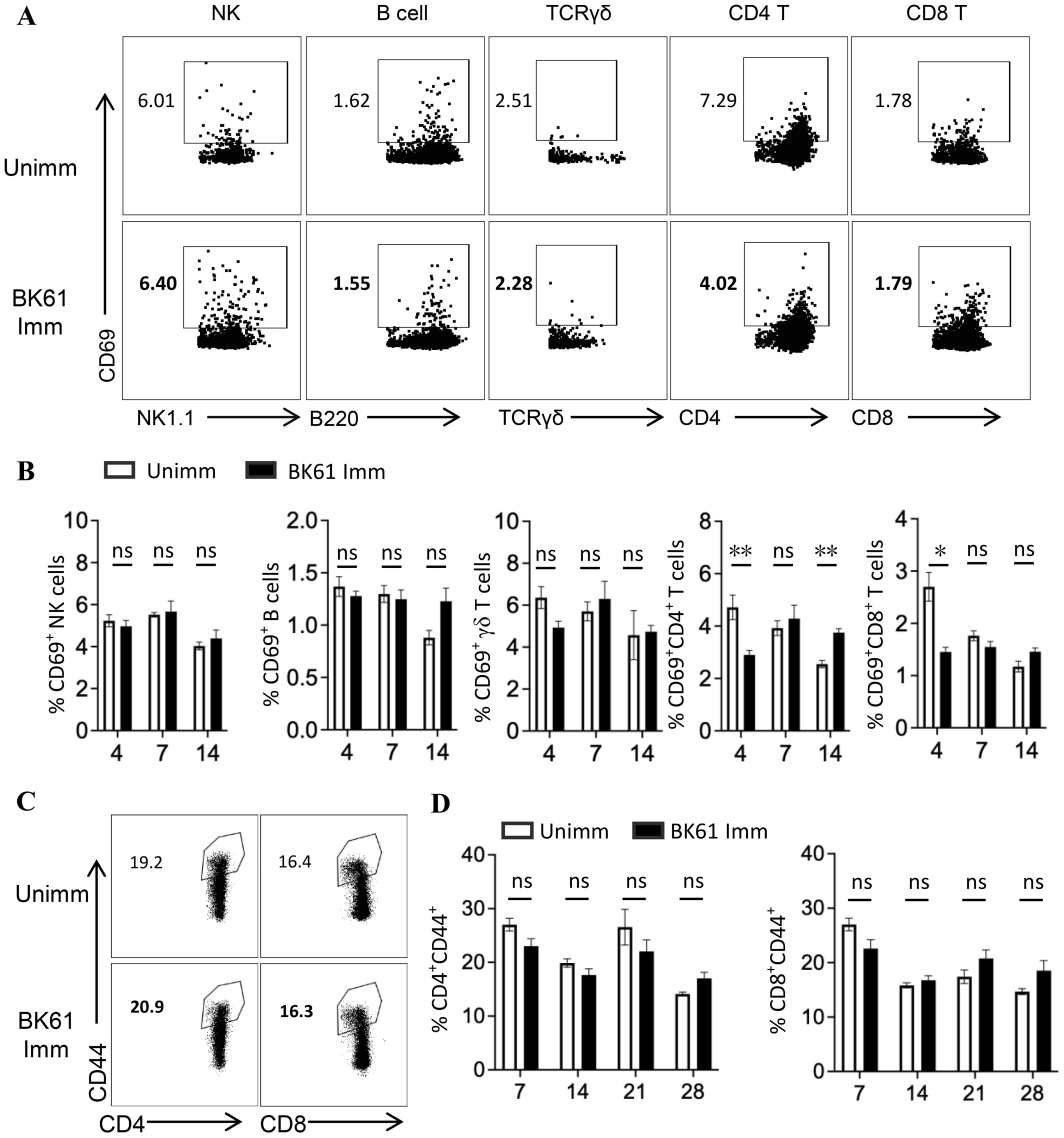
Vaccination with PRV Bartha K61 induce no detectable phenotypic activation of NK cells, B cells and T cells. C57BL/6 mice were immunized with or without PRV Bartha K61 (BK61) at a titer of 10^5^ TCID_50_ and euthanized at indicated time-points, splenocytes were prepared for phenotypic analysis by flow cytometry. **(A)** Representative dot-plots depict CD69 expression on NK cell, B cell, γδ T cell, CD4 and CD8 T cells from unimmunized (upper row) and immunized mice (lower row) at 7 dpi. Numbers indicate the percentages of CD69-positive cells. **(B)** Kinetic changes of the percentages of CD69-positive NK cells, B cells, γδ T cells, CD4 and CD8 T cells in the spleen of unimmunized and BK61-immunized mice. **(C)** Representative dot-plots of CD44^hi^ CD4 and CD8 T cells from unimmunized and immunized mice at 14 dpi. Numbers indicate the percentages of CD44^hi^-positive T cells. **(D)** Kinetic changes of the percentages of CD44^hi^ CD4 and CD8 T cells in the spleen of unimmunized and BK61-immunized mice. Data shown are mean ± SD from four mice per group. ns, no statistical significance; *P < 0.05, **P < 0.01.

Attenuated live vaccines was shown to elicit potent cellular immune responses in mice or human after immunization that significant increase of CD44^hi^CD62L^-^, CD44^hi^CD62L^+^ or IFN-γ-producing T cells was observed (9, 37–39). Therefore, we anticipated that vigorous activation of distinct lymphocytes should occur after PRV LAV vaccination. Thus, we detected the expression of CD69 and CD44 on lymphocyte subsets to evaluate the very early activation and antigen-experienced effector and memory T cells in mice after immunization (40, 41). Surprisingly, vaccination with PRV Bartha K61 (BK61) did not induce the upregulation of CD69 on NK cells, B cells, γδ T cells, and CD4 and CD8 T cells at day 4, 7 and 14 after immunization (dpi) (**Figures 1A, B**). Instead, PRV BK61 immunization even led to the reduction of CD69-positive CD4 and CD8 T cells at 4 dpi (**Figure 1B**). To further confirm if there is late activation of T cells or memory T cell development, we detected the CD44 expression on T cells. As shown in **Figures 1C, D**, PRV BK61 immunization did not increase the percentage of CD44^hi^-postive effector and memory CD4 and CD8 T cells at any time-point after vaccination, compared to the un-immunized mice. These results suggested that PRV BK61 induced no detectable phenotypic activation of NK cells, B cells and T cells in mice.

### PRV BK61 immunization induced antigen-specific CD4 and CD8 T cells at low frequency

**Figure 2:**
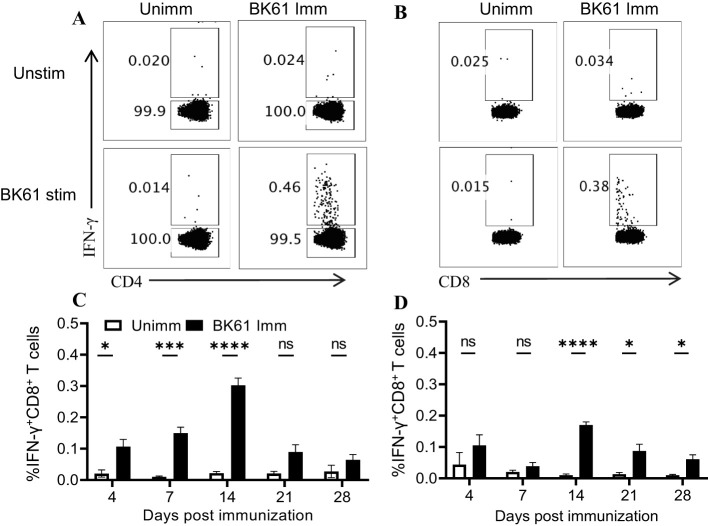
PRV BK61 immunization induce antigen-specific CD4 and CD8 T cells at low frequency. Splenocytes were prepared from PRV BK61-immunized and unimmunized C57BL/6 mice at indicated time-points after immunization and restimulated with or without live BK61 for 18 hours and followed by intracellular staining of IFN-γ and analyzed by flow cytometry. **(A, B)** Representative dot-plots of IFN-γ-producing CD4 and CD8 T cells from unimmunized (left) and immunized mice (right) at 14 dpi, respectively. Numbers indicate the percentages of IFN-γ-positive T cells. **(C, D)** Kinetic changes of the percentages of IFN-γ-positive CD4 and CD8 T cells in the spleen of unimmunized and BK61-immunized mice at 4, 7, 14, 21 and 28 dpi. Data shown are mean ± SD from four mice per group. ns, no statistical significance; *P < 0.05, ***P < 0.001, ****P < 0.0001.

To further determine whether PRV BK61 vaccination induce PRV-specific T cells response, we examined the frequency of IFN-γ-producing T cells upon ex vivo restimulation of the BK61-primed T cells with live BK61 through intracellular cytokine staining (ICS). As shown in **Figures 2A, B**, we detected more IFN-γ-producing CD4 and CD8 T cells in the BK61-immunized mice, compared to the unimmunized control, but the frequencies of both IFN-γ^+^CD4^+^ and IFN-γ^+^CD8^+^ T cells were very low (less than 0.5%). Further analysis of the kinetics of PRV-specific T cells showed that both IFN-γ^+^CD4^+^ and IFN-γ^+^CD8^+^ T cells peaked at 14 dpi in terms of percentage but the magnitude of IFN-γ^+^CD4^+^ T cells was higher than that of IFN-γ^+^CD8^+^ T cells and PRV-specific CD4 T cells were detected earlier (4 dpi) than PRV-specific CD8 T cells (**Figure 2B**). These results indicated that PRV BK61 induced a dominant CD4 Th1 response in mice with low magnitude.

### PRV BK61-primed CD4 but not CD8 T cells exhibit characteristics of multi-functional T cells

**Figure 3:**
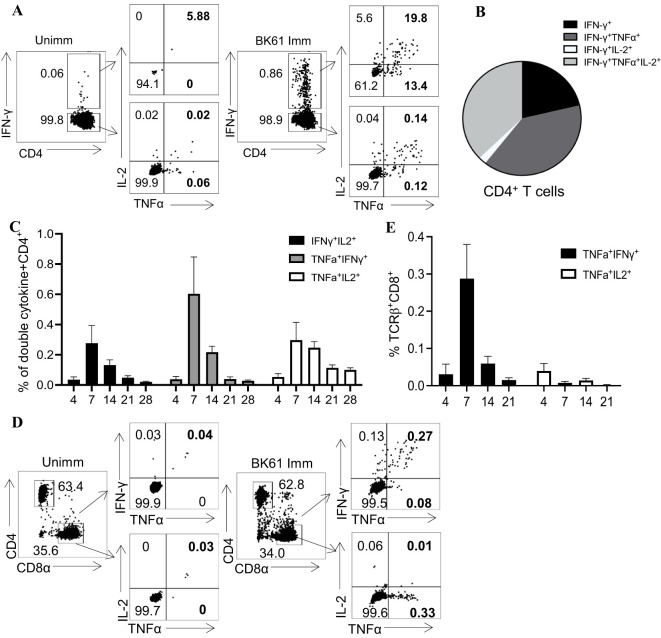
PRV BK61-primed CD4 but not CD8 T cells exhibit characteristics of multi-functional T cells. Splenocytes from unimmunized and BK61-immunized C57BL/6 mice were stimulated *ex vivo* for 18h with live BK61 at MOI of 1 at indicated time-points after immunization and then harvested for intracellular staining of IFN-γ, TNF-α and IL-2. **(A)** Representative dot-plots of multiple cytokine-producing CD4 T cells in the spleens of unimmunized and immunized mice at 14 dpi. **(B)** Pie graphs show relative proportion of TNF-α^+^, IL-2^+^ and TNF-α^+^IL-2^+^ CD4^+^ T cells among IFN-γ^+^CD4^+^ T cells from the spleens of immunized mice at 14 dpi. **(C)** The changes of percentage of PRV-specific double-cytokine (IFN-γ^+^TNF-α^+^, IFN-γ^+^IL-2^+^ and TNF-α^+^IL-2^+^)-producing CD4^+^ T cells detected in BK61-immunized mice at different time-points after immunization. **(D)** Representative dot-plots of double cytokine (IFN-γ^+^TNF-α^+^, TNF-α^+^IL-2^+^)-producing CD8 T cells in the unimmunized and immunized mice at 14 dpi. **(E)** The changes of percentage of PRV-specific double-cytokine (IFN-γ^+^TNF-α^+^ and TNF-α^+^IL-2^+^)-producing CD8 T cells detected in BK61-immunized mice after immunization. Data shown are mean ± SD from four mice per group.

Among activated T cells, polyfunctional T cells that are able to simultaneously produce multiple cytokines (IFN-γ, TNF-α and IL-2) have been shown to correlate with protective immunity, and are equivalent to central memory T cells (42, 43). We further analyzed the functionalities of those PRV-specific T cells by ICS of multiple cytokines though their frequency was low. As shown in **Figures 3A, B**, compared to the unimmunized mice, BK61-immunized mice have more PRV-specific IFN-γ-producing CD4 T cells, which also simultaneously co-expressed TNF-α (IFN-γ^+^TNF-α^+^) or IL-2 (IFN-γ^+^IL-2^+^) or TNF-α and IL-2 (IFN-γ^+^TNF-α^+^IL-2^+^), with higher proportion in IFN-γ single positive (61.2%) and IFN-γ^+^TNF-α^+^IL-2^+^ CD4 T cells (19.8%) (**Figures 3A, B**). In addition, there were also slightly more IFN-γ-negative TNF-α^+^, IL-2^+^ and TNF-α^+^IL-2^+^ CD4 T cells in BK61-immunized mice (**Figure 3A**). These results demonstrated that BK61 immunization induced multi-functional CD4 T cells. Further analysis of the kinetics of PRV-specific CD4 T cells showed that all three subsets of double-cytokine-producing CD4 T cells peaked at 14 dpi and then decreased by 28 dpi (**Figure 3C**). In contrast, very few of IFN-γ^+^TNF-α^+^CD8 T cells were induced in BK61-immunized mice. These cells showed similar kinetics to that of cytokine-producing CD4 T cells but no triple cytokine-producing CD8 T cells were detected after immunization (**Figures 3D, E**). These results showed that PRV BK61-primed CD4 but not CD8 T cells showed characteristics of multi-functional T cells.

### PRV-specific CD4 T cells showed weak memory response upon challenge with PRV DX strain

**Figure 4:**
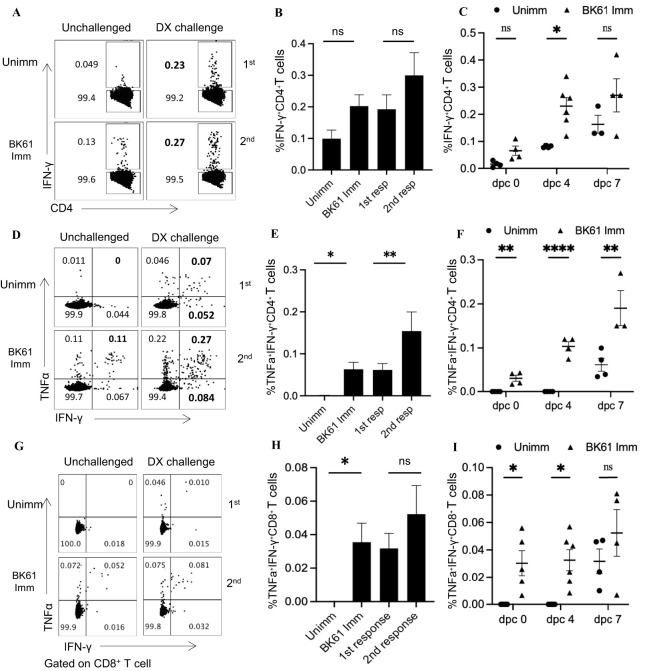
PRV-specific CD4 T cells showed weak memory response upon challenge with PRV DX strain. C57BL/6 mice were immunized with or without PRV Bartha K61 and rested for 28 days, then the immunized and age-matched naïve mice were challenged with or without PRV DX strain. The primary and secondary response of IFN-γ- or IFN-γ and TNF-α-producing CD4 and CD8 T cells in the naïve and prior immunized mice were compared by flow cytometry. **(A)** Representative dot-plots of IFN-γ-positive CD4 T cells in the spleens of indicated mice challenged with or without DX strain. Numbers indicate the percentages of IFN-γ^+^CD4^+^ T cells. **(B)** Bar graph shows the percentages of IFN-γ^+^CD4^+^ T cells in the spleens of indicated mice at 7 days post-challenge (dpc). **(C)** The kinetic changes of the percentages of primary and secondary IFN-γ^+^CD4^+^ T cells from the naïve and previously immunized mice at 0, 4 and 7 dpc. **(D)** Representative dot-plots of IFN-γ^+^TNF-α^+^CD4 T cells in the spleens of indicated mice challenged with or without DX strain. **(E)** The comparison of the percentages of IFN-γ^+^TNF-α^+^CD4 T cells in the spleens of indicated mice at 7 dpc. **(F)** The kinetic changes of the percentages of primary and secondary IFN-γ^+^TNF-α^+^CD4 T cells from the naïve and previously immunized mice at 0, 4 and 7 dpc. **(G)** Representative dot-plots depict IFN-γ^+^TNF-α^+^CD8 T cells in the spleens of indicated mice challenged with or without DX strain. **(H)** The comparison of the percentages of IFN-γ^+^TNF-α^+^CD8 T cells in the spleens of indicated mice at 7 dpc. **(I)** The kinetic changes of the percentages of primary and secondary IFN-γ^+^TNF-α^+^CD8 T cells from the naïve and previously immunized mice at 0, 4 and 7 dpc. Data shown are mean ± SD from four mice per group. ns, no statistical difference, *P < 0.05, **P < 0.01, ****P < 0.0001.

It has been reported that PRV vaccine-induced T-cell immunity played a critical role in early protection against PRV in pigs and repeated vaccination are required clinically for long-term protection (28, 29), suggesting that PRV attenuated vaccine may induce weak memory T cells response. Therefore, we examined the secondary response of PRV-specific T cells after challenge with PRV DX strain. Both BK61-immunized and unimmunized mice were challenged at 28 dpi or left unchallenged and IFN-γ- and TNF-α-producing T cells were detected at 7 dpc upon restimulation with BK61. As shown in **Figure 4**, when comparing the percentage and kinetic change of total IFN-γ-producing CD4 T cells, there was no significant difference between the immunized and unimmunized mice after DX challenge (**Figures 4A–C**). However, when comparing the percentage of IFN-γ^+^TNF-α^+^ CD4 T cells, BK61-immunized mice have more IFN-γ^+^TNF-α^+^ CD4 T cells than the unimmunized mice at 4 and 7 days after DX challenge (**Figures 4D–F**), which were detected as early as 4 dpc (**Figure 4F**), suggesting BK61 immunization had induced antigen-specific memory CD4 T cells, thus have rapid secondary response after challenge. Similarly, we also analyzed BK61-induced memory CD8 T cells by comparing the percentage of IFN-γ^+^TNF-α^+^ CD8 T cells between the two groups after challenge. We found no significant increase in secondary CD8 T cells response, compared to primary CD8 T cells response in terms of percentage (**Figures 4F–H**). Of note, the percentages of both PRV-specific secondary CD4 and CD8 T cells were very low though BK61-primed T cells were pre-existed before challenge. These results indicated that BK61 immunization mainly induced a weak memory response of CD4 T cells.

### Vaccination with BK61-infected dendritic cells transiently enhanced PRV-specific T cell response but not protective efficacy

**Figure 5:**
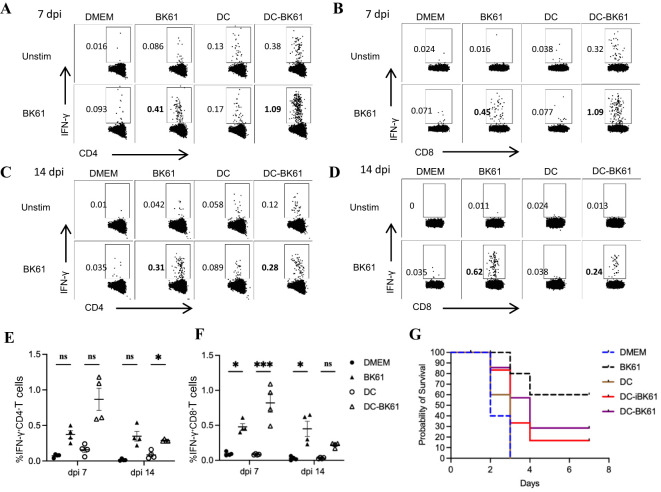
Immunization with BK61-infected dendritic cells transiently enhanced PRV-specific T cell response but not protective efficacy against PRV DX challenge. C57BL/6 mice were immunized with DMEM, PRV Bartha K61, bone marrow-derived dendritic cells (DCs) and BK61-pulsed DCs, respectively, and euthanized at 7 and 14 days after immunization or challenged with PRV DX strain at 28 dpi. Splenocytes from each group were prepared at indicated time-points and restimulated with or without live BK61 for 18 hours and followed by intracellular staining of IFN-γ and analyzed by flow cytometry. Survival rates of each group were recorded after PRV DX challenge. **(A–D)** Representative dot-plots of PRV-specific IFN-γ-producing CD4 **(A, C)** and CD8 T cells **(B, C)** in the spleens of indicated mice at 7 **(A, B)** and 14 days **(C, D)** after vaccination. **(E, F)** The kinetic changes of the percentages of IFN-γ-producing CD4 **(E)** and CD8 **(F)** T cells from the spleens of mock-, BK61-, DC only- and DC-BK61-immunized mice at 7 and 14 dpi. **(G)** The survival curves of mock-, BK61-, DC only- and DC-BK61-immunized mice after PRV DX challenge. Data shown are mean ± SD from four mice per group. ns, no statistical difference, *P < 0.05, ***P < 0.01.

Previous studies showed that antigen-specific CD4 and CD8 T cells response can be rapidly induced and potently enhanced by dendritic cell (DC) immunization (33, 44). To further demonstrate whether the weak T cells response induced by BK61 is an intrinsic property of PRV BK61. We employed DC immunization to enhance BK61-induced T cells response. BMDCs were pulsed with live or inactivated PRV BK61 at MOI of 0.5 (DC-BK61 or DC-iBK61) and then used to immunize mice. PRV-specific CD4 and CD8 T cells were examined and compared after immunization as aforementioned protocol. As shown in **Figures 5A, B**, PRV-specific IFN-γ-producing CD4 and CD8 T cells response were significantly enhanced at 7 dpi by DC-BK61 immunization as DC-BK61-immunized mice have the highest percentage (1%) of IFN-γ-producing CD4 and CD8 T cells, compared to the other three groups (DMEM, DC alone and BK61). However, by 14 dpi, this difference was no longer observed between BK61 and DC-BK61 group (**Figures 5C, D**). To further test whether DC-BK61-enhanced T cell response was translated into better protection against PRV DX challenge. The survival rates of each group were compared after challenge. As a result, DC vaccination with BK61 or inactivated BK61 did not provide better protection than BK61 immunization (**Figure 5F**). These results indicated that although the weak T cells response induced by BK61 were transiently enhanced by dendritic cell vaccination, the eventual protective immunity was not increased, suggesting that there is an intrinsic drawback for PRV BK61 in inducing cellular immunity to some extent.

### The weak Th1-biased immunity may be an intrinsic property of PRV LAVs

**Figure 6:**
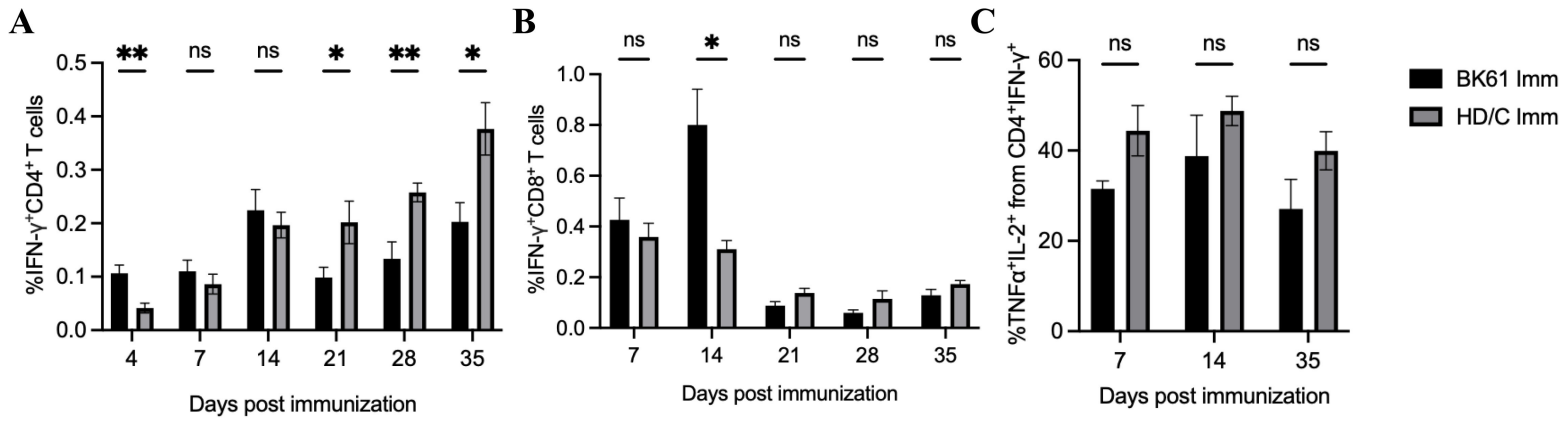
Low magnitude of antigen-specific CD4 and CD8 T cells were induced by another PRV attenuated strain HD/c in mice. C57BL/6 mice were immunized with PRV Bartha K61 and HD/c, respectively. Splenocytes from both groups were prepared at indicated time-points after immunization and restimulated with or without live BK61 or HD/c strain, respectively, for 18 hours and followed by intracellular staining of IFN-γ and compared by flow cytometry. The percentages of IFN-γ^+^CD4 T cells **(A)**, IFN-γ^+^TNF-α^+^IL-2^+^CD4 T cells **(B)** and IFN-γ^+^CD8 T cells **(C)** from the spleens of BK61- and HD/c-immunized mice (n=8) were compared at indicated time-points after immunization. Data shown are mean ± SEM pooled from eight mice from two independent experiments. ns, no statistical difference, *P < 0.05, **P < 0.01.

**Figure 7:**
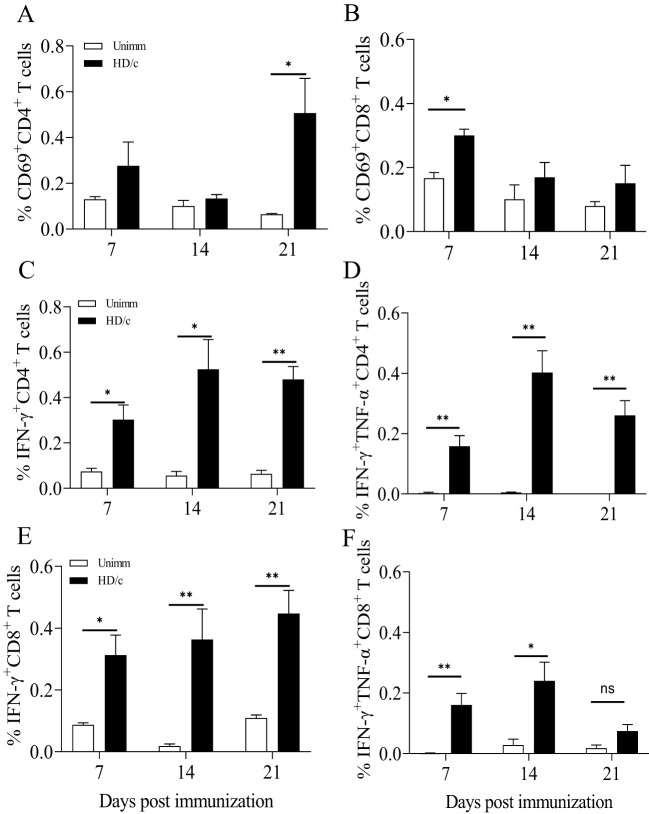
The attenuated PRV vaccine also induced a weak Th1-biased immunity in pigs. Six-week-old piglets were immunized with or without PRV HD/c and PBMCs from the unimmunized and immunized pigs were isolated at indicated time-points after immunization. The expression of CD69 and IFN-γ and/or TNF-α by CD4 and CD8 T cells were detected by flow cytometry. **(A, B)** Representative dot-plots depict CD69 expression on CD4 **(A)** and CD8 **(B)** T cells from the unimmunized and immunized pigs at 7, 14 and 21 dpi. Numbers indicate the percentages of CD69-positive cells. **(C, D)** The kinetic changes of the percentages of IFN-γ-positive **(C)** or IFN-γ and TNF-α double-producing CD4 T cells **(D)** in the PBMCs of the unimmunized and immunized pigs upon ex vivo restimulation with live HD/c at 7, 14, 21 dpi. **(E, F)** The kinetic changes of the percentages of IFN-γ-positive **(E)** or IFN-γ and TNF-α double-producing CD8 T cells **(F)** in the PBMCs of the unimmunized and immunized pigs upon ex vivo restimulation with live HD/c at 7, 14, 21 dpi. Data shown are mean ± SD from four pigs per group. ns, no statistical significance; *P < 0.05, **P < 0.01.

PRV BK61 strain originally contains deletions of the US2, gE, gI, and US9 genes. To test whether other gene-deleted attenuated PRV strain also induce similar T cell responses in mice, we examined PRV-specific IFN-γ-producing T cells elicited by PRV HD/c strain, a gE/TK-deleted PRV variant strain constructed in our lab (21). As shown in **Figure 6**, PRV HD/c immunization induced similar kinetics and magnitude of IFN-γ-producing CD4 and CD8 T cells as well as IFN-γ^+^TNF-α^+^IL-2^+^CD4 T cells before 14 dpi and more IFN-γ^+^CD4 T cells from 21 to 35 dpi, compared to the BK61 immunization, suggesting that the magnitude of T cell response (less than 0.5%) may be determined by the PRV LAVs itself but not by the difference of gene deletions in the LAVs. To further confirm whether attenuated PRV vaccine induce similar magnitude of T cells responses in pigs as well, we used HD/c to immunize piglets and examined the kinetics and magnitude of IFN-γ-expressing T cells in PBMCs of the immunized pigs. As shown in **Figure 7**, HD/c immunization barely induced phenotypic activation (CD69^+^) of CD4 T cells at 21 dpi and CD8 T cells at 7 dpi with the percentages less than 0.6% in the PBMCs (**Figures 7A, B**). PRV-specific IFN-γ^+^ or IFN-γ^+^TNF-α^+^ CD4 T cells and CD8 T cells were significantly elicited in the PBMCs of pigs after PRV HD/c immunization but with low frequency (less than 0.6%) as well (**Figures 7C–F**). No PRV-specific triple cytokine-producing T cells were detected in the immunized pigs (data not shown). These results demonstrated that attenuated PRV vaccine strain also induced weak Th1 immunity in pigs, similar to that observed in mice, which may be an intrinsic property of PRV LAVs.

## Discussion

Aujeszky’s disease caused by PRV has substantial impact on the pig industry in China (1). Although this disease has been controlled by the use of vaccination with PRV LAVs, especially the Bartha K61 (BK61) strain (7), the magnitude and kinetics of PRV BK61-induced cellular immunity has not been fully examined. In this study, through a comprehensive analysis of the phenotypic and functional changes and the magnitude of PRV-specific T cell response *in vivo* after vaccination, we found that PRV BK61 induced a weak cellular immunity in mice and pigs, phenotypically manifested by undetectable T cell activation and expansions in terms of CD69 and CD44 expression, low frequency of PRV-specific CD4 T cells that can be temporarily enhanced by a strategy of DC immunization, suggesting that this might be the intrinsic property or drawbacks of PRV LAVs.

Modified live virus vaccines are generally believed to elicit potent cellular immune responses and antibody responses (9, 37–39). For instance, influenza LAV (DelNS1-LAIV) and a mouse coronavirus LAV (N7-MTase-mutated MHV) induced significant increase of effector and memory T cells after challenge (37) and about 2% IFN-γ-producing T cells in mice (38). It was surprisingly unexpected that PRV BK61 induced such a weak cellular immunity in this study. Initially, we directly compared the change of T cell frequency before and after BK61 immunization, but no difference was observed. Therefore, we chose to detect the very early activation marker CD69 (40) and antigen-experienced T cells marker CD44 (41), but still no apparent activation of T cells was observed (**Figure 1**). Lastly, we detected PRV-specific IFN-γ-producing T cells by *in vitro* stimulation with live BK61. Indeed, we successfully detected PRV-specific CD4 and CD8 T cells, but their percentages are unexpectedly low as well (less than 0.5%) (**Figure 2**). Live BK61 was supposed to encode many antigens that were presented to all BK61-primed T cells to stimulate them producing cytokines during *in vitro* co-culture. To increase the detection rate of IFN-γ-producing T cells, we had tried to use BK61-pulsed BMDCs as stimuli for intracellular cytokine staining assay because antigen-pulsed BMDCs have been shown to be able to stimulate the primed T cells to produce more IFN-γ (36). However, we still cannot detect higher percentage of PRV-specific IFN-γ-producing T cells (data no shown). These results indicate that PRV BK61 immunization truly induce a low magnitude of PRV-specific T cells response. In addition, when comparing the percentage of PRV-specific IFN-γ^+^CD4^+^ T cells, no memory response was detected after challenge (**Figures 4A–C**), only a small portion of IFN-γ^+^TNF-α^+^ CD4 T cells showed significant secondary response after challenge (**Figures 4D–F**), suggesting that PRV BK61-primed T cells truly have a weak memory response. Of note, PRV HD/c also induced a low magnitude of PRV-specific T cells response as did BK61 (**Figure 6**) even though PRV HD/c was inoculated at a high dose compared to BK61 (10^7.25^ VS 10^5.0^ TCID_50_). All these results implied that PRV BK61-induced weak Th1-biased immunity is likely an intrinsic property of PRV LAVs.

DC immunization has shown great potency in enhancing CD4 and CD8 T cells response and immune protection (33, 44). In this study, BK61-BMDC vaccination indeed enhanced BK61-specific IFN-γ-producing CD4 and CD8 T cells at 7 dpi but did not improve the protective efficacy after DX challenge (**Figure 5**). The reason behind this might be that the cellular immunity elicited by BK61-DC immunization was not strong enough or long-lasting. Attenuated PRV BK61 was shown to be neurotropic in mice and can transmit to the PNS via sensory and motor neuronal pathways (45). It was reported that PRV virion captured cellular kinesin motor in epithelial cells for its subsequent invasion of neuronal cells (46). If so, once DC-carrying PRV BK61 gets into the body, the virus may quickly and eventually infect neuronal cells, leading to insufficient antigen presentation by APCs to prime T cells. This may also explain why attenuated PRV induces such a weak cellular immune response. But further investigation is needed.

Although PRV-specific IFN-γ-producing memory CD4 T helper cells and CD8 CTL had been detected in DBA/2 mice and pigs in previous studies (22, 25–27), most of those findings were from *in vitro* long-term culture and enrichment of lymphocytes upon restimulation with BK61 or lymphoproliferative assay. The kinetic changes and magnitude of PRV-specific T cells were lacking after immunization. In this study, through multicolor flow cytometry, we demonstrated that PRV BK61 mainly induced a CD4 Th1-biased response in both mice and pigs that peaked at 14 dpi with low frequency (**Figures 2**, **7**) and IFN-γ^+^CD8 T cells seemed to play a dispensable role, consistent with a previous study (30). As previous studies showed that the magnitude of the IFN-γ response in PBMCs of pigs was correlated best with protection against virus shedding (28) and *in vivo* depletion of CD4 T cells and IFN-γ led to less protection of the BK61-immunized mice from challenge (30). These results highlighted the importance of IFN-γ-producing CD4 T cells in protective immunity against PRV infection. Of note, due to that PRV-induced Th1-biased immunity is more favorable to help antibody secretion by B cells (27), PRV may not be an ideal viral vector for developing a vectored-vaccine against PRRSV and ASFV whose immune protection are believed to largely rely on cell-mediated immunity (32, 47, 48).

Taken together, through a comprehensive analysis of PRV-specific T cells response, we demonstrated that attenuated PRV vaccine induced a Th1-biased cellular immunity in mice and pigs that is relatively weak, which might be an intrinsic property or drawbacks of PRV LAVs. Our findings provide a rationale for the design of PRV-vectored vaccines and may also be implicative for the selection of other viral vectors for vaccine development.

## Data Availability

The original contributions presented in the study are included in the article/supplementary material. Further inquiries can be directed to the corresponding authors.
